# Genomic prediction of feed efficiency in boars by deep learning

**DOI:** 10.1093/g3journal/jkaf274

**Published:** 2025-11-14

**Authors:** Olumide Onabanjo, Theo Meuwissen, Hans Magnus Gjøen, Fadi Al Machot, Maren van Son, Peer Berg

**Affiliations:** Department of Animal and Aquacultural Sciences, Norwegian University of Life Sciences, 1432 Ås, Norway; Department of Animal and Aquacultural Sciences, Norwegian University of Life Sciences, 1432 Ås, Norway; Department of Animal and Aquacultural Sciences, Norwegian University of Life Sciences, 1432 Ås, Norway; Department of Data Science, Norwegian University of Life Sciences, 1432 Ås, Norway; Norsvin SA, 2317, Hamar, Norway; Department of Animal and Aquacultural Sciences, Norwegian University of Life Sciences, 1432 Ås, Norway

**Keywords:** deep learning, genomic prediction, feed efficiency, complex trait, nonadditive genetic variance

## Abstract

Pork is the most widely consumed meat globally, and the industry has achieved substantial genetic advancements for several traits using genomic selection. However, traditional linear genomic prediction models may be inadequate for predicting complex traits, such as feed efficiency, as they primarily capture additive genetic effects and overlook nonadditive effects, including dominance and epistasis. Deep learning (DL) has the potential to address this limitation due to its ability to model nonlinear patterns inherent in genomic data. The objectives of this study were to compare the predictive ability of DL models to the linear models for predicting feed efficiency (FE) trait in 2 boar populations, estimate the nonadditive genetic variance captured by DL, and assess its effect on predictive ability. Our results showed that the DL models using the averaged-prediction method had the highest predictive ability in the sire line test population (0.381 for multilayer perceptron [MLP] and 0.377 for convolutional neural network [CNN]), compared to 0.366 for linear models. DL models also showed higher abilities in the dam line test population, with MLP achieving a predictive ability of 0.364. Additionally, we showed that DL models captured nonadditive variance; however, this did not significantly improve predictive ability. In conclusion, DL models, particularly MLP, demonstrated the highest predictive ability for FE, improving performance by approximately 4.1% for the sire line and 2.8% for the dam line compared to the traditional linear models. Therefore, DL models are recommended for predicting phenotypes and for estimating total genetic effects, including nonadditive components. However, this comes at a significant increase of computational cost.

## Teaser

The target audience for this research are researchers with interest in using deep learning (DL) for genomic prediction. The article presents a different way of estimating DL prediction accuracy, provides insight into using data augmentation technique in animal breeding, and presents a novel method of estimating genetic variance with DL. In this article, Onabanjo et al. used DL for genomic prediction of feed efficiency (FE). The authors hypothesized that DL models might capture the unknown patterns of gene interactions for complex traits and thus might have more accurate prediction compared to linear models. Their results confirmed that DL models capture non-additive genetic effects.

## Introduction

Pork makes up about 34% of global meat consumption (World Population Review 2025), making it the most widely eaten meat. During the pork production process, feed costs account for approximately 60% to 70% of the total production cost (Patience et al. 2015; Rocadembosch et al. 2016). Therefore, there is a need to breed feed-efficient pigs to maximize farmers' profits, meet the growing demand for pork, and reduce the environmental footprint. The feed efficiency (FE) trait is one of the performance indicators routinely used to measure pig performance and is a complex trait influenced by multiple genes, environment, physiology, and management factors. Genomic selection (Meuwissen et al. 2001) has been used to improve several traits, increase genetic gains, and reduce costs and generation intervals. However, this method has some limitations, including its assumption of a linear relationship between genotypes and phenotypes, while nonadditive genetic effects (dominance and epistasis) resulting from interactions between quantitative trait loci have mainly been ignored (Ehret et al. 2015; Lee et al. 2023).

However, nonadditive genetic effects may also influence the genetic architecture of complex traits (Hill et al. 2008; Vitezica et al. 2017). Some advantages of estimating these nonadditive genetic effects include their contributions to prediction accuracy (Toro and Varona 2010; Duenk et al. 2017), the allocation of mates between selection candidates (Mäki-Tanila 2008; Toro and Varona 2010), and enhancing the use of nonadditive effects in crossbreeding and purebred breeding schemes (Mäki-Tanila 2008). There have been several attempts to extend the genomic prediction model to include dominance effects (Vitezica et al. 2013; Zhao et al. 2015; Varona et al. 2018) and epistasis effects (Wang et al. 2012; Vitezica et al. 2017). However, it becomes computationally expensive and complex beyond pairwise interactions (Jiang and Reif 2015; Chen et al. 2023). However, deep learning (DL) models may have the potential to intrinsically capture these nonadditive genetic effects, as they are nonparametric and have demonstrated the ability to capture nonlinear relationships between predictors and responses (Azodi et al. 2019; Pérez-Enciso and Zingaretti 2019; Costa-Neto et al. 2021).

DL is a subset of machine learning that typically comprises several neural layers, to some extent, inspired by the brain's structure and functioning. Although DL has existed for decades, advances in computing technology and increasing data availability have made it more applicable in recent years (Abdollahi-Arpanahi et al. 2020). It has excelled in different fields, such as computer vision, text, video, and voice interpretation and has also received increased attention for genomic data analysis and prediction of complex traits (Pérez-Enciso and Zingaretti 2019; Montesinos-López et al. 2024). DL algorithms, including the multilayer perceptron (MLP) and convolutional neural networks (CNN), may be able to exploit unknown patterns of gene interactions and linkage disequilibrium (Abdollahi-Arpanahi et al. 2020). Several studies have examined the performance of these algorithms for genomic predictions of various traits in plant breeding (Gianola et al. 2011; González-Camacho et al. 2012, 2016; Pérez-Rodríguez et al. 2012, 2020; Rachmatia et al. 2017; Ma et al. 2018; Montesinos-López et al. 2018; Khaki and Wang 2019; Liu et al. 2019; Pook et al. 2020; Sandhu et al. 2020; Zingaretti et al. 2020) and animal breeding (Ehret et al. 2015; Abdollahi-Arpanahi et al. 2020; Pedrosa et al. 2024; Su et al. 2025; Wang et al. 2025). Although some of these studies reported increased predictive ability with DL, no definite evidence indicates that DL performs better than conventional linear genomic prediction models.

The objectives of this study were (i) to estimate the proportion of additive and nonadditive variances for the FE trait using the conventional linear genomic prediction models, (ii) to evaluate and compare the predictive ability of DL models (MLP and CNN) to the linear genomic prediction models in predicting FE in boars. Additionally, we aimed to determine whether (iii) a data augmentation technique using variational autoencoders (VAE) improves the predictive ability of DL and (iv) to estimate the nonadditive genetic variance captured by DL and assess its effect on predictive ability.

## Materials and methods

### Dataset

#### Genotype data

Topigs Norsvin provided genotypes for approximately 30,000 sire line and dam line boars tested at their testing station between 2011 and 2023. Boars were genotyped using 60k, 50k, or a custom array containing 25k single-nucleotide polymorphisms (SNPs), and genotype imputation was used to achieve a uniform density of ∼50k SNPs for all animals. Quality control (QC) was carried out using Plink (Purcell et al. 2007) with the following criteria: SNPs with minor allele frequency > 0.05, deviations from HWE > 10^−25^, and SNPs located on the sex chromosomes were excluded. This reduced the SNP density for the sire line to 36,313 (73% of the total) and the dam line population to 35,977 (74% of the total) SNPs, as shown in Table 1. The SNPs that passed QC were used to compute relationship matrices, estimate variances, and predict genetic effects. The SNP genotype matrices, with genotypes coded as 0, 1, and 2 for genotypes AA, Aa, and aa, respectively, were used as input for the DL models.

**Table 1.** Summary of the dataset.

	Sire line	Dam line
Number of boars	15,711	13,944
Total number of SNPs	49,478	48,617
Number of SNPs after QC	36,313	35,977
Number of pens	1,515	1,416
Number of litters	9,336	12,011
Average feed intake from 40 to 120 kg body weight	169.236 ± 13.049	169.968 ± 12.346
Average days from 40 to 120 kg body weight	72.479 ± 9.121	72.019 ± 6.819
Average feed efficiency	0.475 ± 0.036	0.473 ± 0.033
Average pre-corrected feed efficiency	3.22e^−5^ ± 0.022	3.18e^−5^ ± 0.022

#### Phenotype data

Topigs Norsvin also provided the dataset of boars' growth and feed consumption data, which were collected for all boars tested at their boar test station. For this research, we utilized phenotype data on feed intake for animals with a live body weight starting at approximately 40 kg and ending at approximately 120 kg. The FE trait was estimated as: $\mathrm{FE}=\frac{\mathrm{weight}\;\mathrm{gain}}{\mathrm{feed}\;\mathrm{intake}}$, where weight gain is the difference between the ending and starting body weight, and feed intake is the total feed consumed in kg during that growth period. The FE values for all boars were then pre-corrected for environmental effects, including month of birth, herd-year birth, parity of dam, herd compartment, and random effects of pen (PE) and litter (LI) using a mixed linear model represented as:


(1) $$FE_{abcdef}=MB_{a}+HYB_{b}+\;PD_{c}+HC_{d}+PE_{e}+\;LI_{f}+\mathrm{Residua}l_{abcdef}$$

The pre-corrected values were then z-score normalized (mean = 0, standard deviation = 1) and used as the phenotype when estimating variances and predicting genetic effects based on SNP markers. Only individuals with both phenotype and genotype data were included in the analysis. The dataset used in this study is summarized in Table 1.

### Statistical and deep learning models

#### Nonlinear deep learning models

**Fig. 1.**
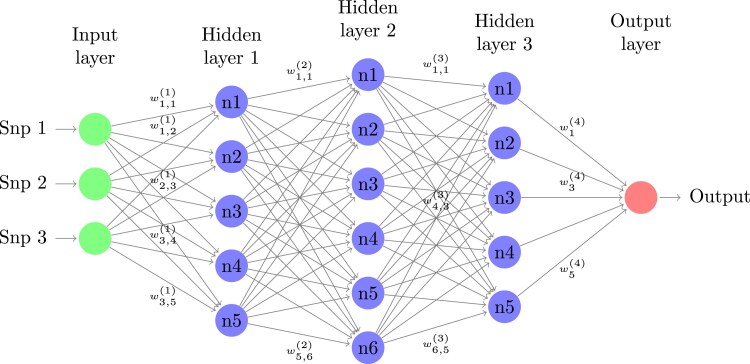
A graphical representation of an MLP network for a regression problem with 3 SNP genotypes as input, 3 hidden layers (5, 6, and 5 neurons, respectively), and one output. The figure was drawn using code from http://www.texample.net/tikz/examples/neural-network.

***Multilayer perceptron:*** A multilayer perceptron consists of the input, the hidden, and the output layers, all fully connected by a dense network of neurons (Angermueller et al. 2016). The input layer accepts a matrix of marker genotypes (X) coded as 0, 1, and 2, which are passed to neurons in the hidden layer(s), where they are multiplied by an initially randomly sampled weight matrix (**W**). Then, a vector of bias (**b**) is added before a nonlinear activation function acts on it, producing an output matrix (H) that serves as an input for the neurons in the next hidden layer, and this process continues through all hidden layers. The matrix representation of the computation going on in a hidden layer is represented as:


(2) $$H^{l}=\;\phi (X^{\left(l-1\right)}W^{l}+\;b^{l}),$$

where *ϕ* represents the leaky ReLu activation function, $X^{\left(l-1\right)}$ is the input genotype matrix from the neurons of the previous layer (*l − 1*), $W^{l}$ is the matrix containing the weights for all neurons in layer *l*, $b^{l}$ is a vector of the bias terms for all neurons in layer *l*, and $H^{l}$ is the output matrix of computations in hidden layer *l*, which serves as the input of neurons in the layer *l + 1*. The inputs receive randomly sampled associated weights and a bias in each neuron of the hidden layer of a network before a nonlinear activation function is applied. The activation functions introduce non-linearity into the network, enabling it to learn and model complex patterns in the data. For instance, the leaky ReLu activation function allows for modelling nonlinear relationships, which is crucial for capturing the underlying structure in complex datasets. Depending on the problem, the output layer might apply a linear or nonlinear activation function. For regression problems as studied here, a linear activation function is used. In contrast, for classification problems, the sigmoid activation function is used for binary predictions, while the SoftMax activation function is used for multi-class predictions. Figure 1 illustrates a simple MLP model. A loss function (mean squared error for regression) compares the predicted and target values during the training process, and the loss is minimized through backward propagation. During backward propagation, the error is backpropagated from the output layer to the input layer, allowing the weights and biases to be updated using a gradient descent algorithm to minimize the loss. This process is repeated until convergence or a predefined number of epochs (a complete pass through the training dataset) is completed. See Pérez-Enciso and Zingaretti (2019) for a more comprehensive description of MLP.

**Fig. 2.**
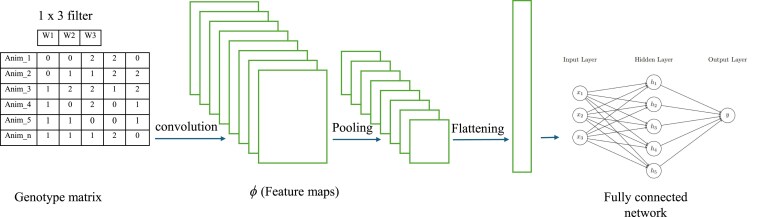
An illustration of the CNN process with an SNP genotype matrix as input. The filter (size = 1 × 3) slides over and multiplies the filter weights by the genotype data of each animal.

***Convolutional neural networks (CNN):*** A convolutional neural network comprises an input layer, a convolution layer(s), a pooling layer(s), a flattening layer, a fully connected layer, and an output layer (Le Cun et al. 1989). In the convolution layer, small matrices called filters slide over the input data, multiplying their weights by the input data and applying an activation function. The filters have shared weights and capture different hidden patterns inherent in the dataset. The patterns or features captured comprise the feature maps, which are then pooled using the average, maximum, or minimum of a defined sequence length. Pooling aims to reduce dimensionality, and after this is done, the features are flattened and passed as inputs to the fully connected neural networks, as shown in Fig. 2. For a more comprehensive description of CNN, see Pérez-Enciso and Zingaretti (2019) and Abdollahi-Arpanahi et al. (2020).

The DL models (MLP and CNN) in this study were implemented in Python v3.11, using functions from Keras v3.3.3 (Chollet 2015), scikit-learn v1.4.2, and TensorFlow v2.16.1 (Abadi et al. 2016). Table 2 provides information about the hyperparameters (the neural network configuration settings) used to train the models, based on the best accuracies obtained after a rigorous hyperparameter tuning process.

**Table 2.** Hyperparameters used to train deep learning models.

	Sire line		Dam line	
	MLP	CNN	MLP	CNN
Number of convolution layers	-	1	-	1
Number of filters	-	16	-	8
Kernel size	-	5	-	5
Pooling size	-	3	-	3
Number of hidden layers	4	3	4	3
Number of neurons per layer	96:64:32:16	64:32:16	96:64:32:16	64:32:16
Kernel initializers	HeNormal	HeNormal	HeNormal	HeNormal
L1 regularizer	0.01	0.005	0.007	0.003
L2 regularizer	0.1	0.1	0.15	0.15
Hidden layers activation function	LeakyReLu	LeakyReLu	LeakyReLu	LeakyReLu
Output layer activation function	linear	linear	linear	linear
Optimizer	Adam	Adam	Adam	Adam
Learning rate	0.00001	0.00001	0.00001	0.00001
Batch size	32	32	32	32
Loss function	MSE	MSE	MSE	MSE

The hyperparameters reported here were selected after a rigorous tuning process.

#### Linear genomic prediction models

The linear genomic prediction models use genomic information from closely related individuals with performance records to predict the genetic merit. This is possible by constructing a genomic relationship matrix (G) using many genome-wide SNP markers. Univariate animal models were fitted following the genome-based restricted maximum likelihood approach in GCTA (Yang et al. 2011) to estimate variance components and calculate genetic values. The additive (A) model or genomic best linear unbiased prediction (GBLUP) was extended to include the nonadditive random effects of dominance (D) and second-order additive-by-additive epistatic interaction (E), resulting in the following 4 models:


(3) $$A\;\mathrm{model}:y=\mu +Z_{1}a+\varepsilon ,$$

(4) $$\mathrm{AD}\;\mathrm{model}:y=\mu +Z_{1}a+\;Z_{2}d+\varepsilon ,\;$$

(5) $$\mathrm{AE}\;\mathrm{model}:y=\mu +Z_{1}a+Z_{3}e_{\mathrm{aa}}+\varepsilon ,$$

(6) $$\mathrm{ADE}\;\mathrm{model}:y=\mu +Z_{1}a+\;Z_{2}d+Z_{3}e_{\mathrm{aa}}+\varepsilon ,$$

where **y** is a vector of pre-corrected phenotypes, μ is the population mean, **a** is a vector of additive genetic effects, **d** is a vector of random dominance effects, $e_{\mathrm{aa}}$ is a vector of the second-order additive by additive epistatic effect, ε is a vector of random residual errors and $Z_{1}$, $Z_{2}$, and $Z_{3}$, are corresponding design matrices with dimensions assigning observations to their vector of random effects. The random effects follow a normal distribution assumption of mean zero and variance:


(7) $$\mathrm{Var}\;\left[\begin{array}{c} a\\ d\\ e_{\mathrm{aa}}\\ \varepsilon \end{array}\right]\;=\;\left[\begin{array}{cccc} G\sigma_{a}^{2} & 0 & 0 & 0\\ 0 & D\sigma_{d}^{2} & 0 & 0\\ 0 & 0 & E_{\mathrm{aa}}\sigma_{e_{aa}}^{2} & 0\\ 0 & 0 & 0 & I\sigma_{\varepsilon }^{2} \end{array}\right],$$

where $\sigma_{a}^{2}$ is the additive genetic variance, $\sigma_{d}^{2}$ is the dominance variance, $\sigma_{e_{aa}}^{2}$ is the epistatic variance, $\sigma_{\varepsilon }^{2}$ is the residual variance, I is an identity matrix, G, D, and $E_{\mathrm{aa}}$ are the additive, dominance, and additive-by-additive genomic relationship matrices.

The phenotypic variance was calculated by summing up all variance components as: $\sigma_{p}^{2}$  *=*  $\sigma_{a}^{2}$  *+*  $\sigma_{d}^{2}$  *+*  $\sigma_{e_{aa}}^{2}$  *+*  $\sigma_{\varepsilon }^{2}$. The variance explained by additive, dominance, and epistasis variations relative to phenotypic variance is defined as narrow-sense heritability ($h^{2}$) = $\frac{\;\sigma_{a}^{2}}{\sigma_{p}^{2}}$, dominance ratio ($d^{2}$) = $\frac{{\;\sigma }_{d}^{2}}{\sigma_{p}^{2}}$, and epistasis ratio ($e_{\mathrm{aa}}^{2}$) = $\frac{\sigma_{e_{aa}}^{2}}{\sigma_{p}^{2}}$. Broad sense heritability ($H^{2}$) was calculated by summing up these variance components $h^{2}$ + $d^{2}$ + $e_{\mathrm{aa}}^{2}$.

### Calculating relationship matrices

#### *Additive genomic relationship matrix*  G

The genomic relationship matrix was constructed using the “–make-grm” function in GCTA (Yang et al. 2011), which implements the method equivalent to VanRaden (2008) method 1:


(8) $$G_{\mathrm{ij}}=\;\frac{1}{\sum_{k=1}^{m}\;2p_{k}(1-\;p_{k})}\overset{m}{\underset{k}{\sum }}\;(t_{ik}-2p_{k})(t_{\;jk}-2p_{k}),$$

where $G_{\mathrm{ij}}$ is the genomic relationship between individuals *i* and *j*, $t_{ik}$ is the genotype of individual *i* at SNP *k*, coded as 0, 1, 2, corresponding to the number of copies of the reference allele, $p_{k}$ is the allele frequency of the reference allele at SNP *k* in the population, and *m* is the total number of SNPs used in the calculation.

#### *Dominance genomic relationship matrix*  D

The dominance relationship matrix was computed using the “–make-grm-d” function in GCTA (Yang et al. 2011), which implements the method described by Zhu et al. (2015):


(9) $$D_{\mathrm{ij}}=\frac{1}{m}\overset{m}{\underset{k=1}{\sum }}\frac{\left(z_{ik}-\;2p_{k}q_{k})(z_{\;jk}-2p_{k}q_{k}\right)}{2p_{k}^{2}q_{k}^{2}},$$

where $D_{\mathrm{ij}}$ is the dominance relationship between individuals *i* and *j*, $z_{ik}$ represents the dominance genotype coding ($z_{ik}=1$ if the genotype is heterozygote Aa and $z_{ik}=0$ if the genotype is homozygote AA or aa) for individual *i* at SNP *k*, $p_{k}$ is the allele frequency of the reference allele at SNP *k*, $q_{k=}\;1-\;p_{k}$, and *m* is the total number of SNPs used in the calculation.

#### *Epistatic genomic relationship matrix*  $E_{\mathrm{aa}}$

This was calculated following Vitezica et al. (2017). The matrix was derived from the Hadamard product operation on the additive genomic relationship matrix represented as:


(10) $$E_{\mathrm{aa}}=\frac{G\;⊙\;G}{v},$$

where **G** is the additive genomic relationship matrix, ⊙ is the Hadamard product operator, and v, which is the scaling factor, is the average of the matrix trace represented as v=tr(G⊙G)/n.

### Model validation and performance assessment

DL models were validated using a 10-fold cross-validation method similar to that used by González-Camacho et al. (2012) and Pérez-Rodríguez et al. (2012). This was repeated 5 times to generate 50 random training and validation dataset splits. The training and validation set included animals born between 2011 and 2021, while the test set consisted of younger animals born in 2022 and 2023. The validation set evaluated the model's performance during training and initiated early stopping, preventing overfitting. The linear models only required training and test sets; no cross-validation was carried out. Table 3 presents the number of boars used for training, validation, and testing the linear and DL models.

**Table 3.** Number of training, validation, and testing individuals of both lines for linear and deep learning models.

Method	Parameter	Sire line	Dam line
Linear	Training set	13,620	11,969
	Validation set	-	-
	Test set	2,091	1,975
	Total	15,711	13,944
DL	Training set	10,896	9,575
	Validation set	2,724	2,394
	Test set	2,091	1,975
	Total	15,711	13,944

Note that both linear and DL models were trained on the same amount of data; however, DL models utilized part of the training data for validation to prevent overfitting.

The performance of the DL models was evaluated using the conventional splits-average approach and the averaged-predictions approach we introduced. This is similar to the bootstrap aggregating (bagging) ensemble method. For the splits-average approach, the Pearson correlation coefficient (*r*) was calculated between the predicted and test phenotypes for each of the 50 splits. Then, the *r* from all 50 splits were averaged to determine the model's split-average predictive ability. In contrast, the averaged-predictions approach introduced was calculated by averaging the predicted values for each test animal across all 50 random splits before calculating *r* between the averaged predicted values and the test phenotypes.

The performance of the linear genomic prediction models was assessed by calculating *r* between the estimated breeding values and the test phenotypes. Note that we herein refer to *r* as predictive ability. The predictive ability and standard error of prediction for linear models were calculated using the formula:


(11) $$r=\mathrm{corr}\;(ebv,\;y_{test}),$$

(12) $$SE_{r}=\;\sqrt{\frac{1-\;r^{2}}{n_{test}\;-\;2}}\;,$$

where *r* is the Pearson correlation coefficient, ebv is the estimated breeding value, $y_{test}$ is the test phenotype, *SE_r_* is the standard error of correlation, and $n_{test}$ is the number of test animals.

### Data augmentation with variational autoencoders

Data augmentation is a widely used technique in DL to generate synthetic data by transforming existing data. The method is used for several reasons, including mitigating the effect of data imbalance, increasing training data size, increasing diversity, reducing overfitting, and improving model performance, robustness, and generalization ability (Islam et al. 2024). Various data augmentation methods exist, such as zooming, cropping, rotating, color jittering, noise introduction, random sampling, and minority oversampling. These methods have been used in different domains, such as computer vision and natural language processing, to improve the performance of DL models. Very few data augmentation methods can be applied to genomic data; therefore, we used the DL-based VAE method to generate meaningful and task-specific data.

VAEs are unsupervised learning artificial neural network architectures that can synthesize new data samples that are different from, yet similar to, the input data on which they are trained. This generative model achieves this by learning and sampling from the latent space in the input data on which they are trained. They have been widely used in generating texts, images, audio, and videos. The VAE architecture comprises 2 distinct networks: an encoder and a decoder. The encoder encodes the essential features of the raw input data into a lower-dimensional latent space represented by a probability distribution from which the decoder samples, thereby synthesizing new samples.

**Fig. 3.**
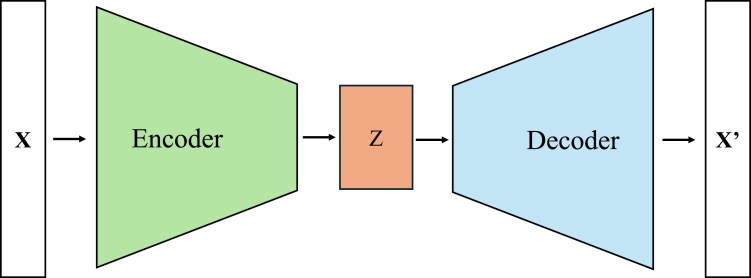
An illustration of the VAE workflow.

Using the VAE, we augmented the boar datasets (**X**) provided by Topigs Norsvin by generating 1000 synthetic animals (**X^’^**) for each line. To accomplish this, the training individuals of each line were divided into 10 bins based on their phenotype distribution. The binned dataset was then supplied to the VAE encoder, which transformed the data into a lower-dimensional (Z) continuous 16-neuron latent space representation. The decoder reconstructed 100 synthetic observations for each bin from this latent space, and the phenotype of each synthetic observation was assigned as the average of the edges of the bin to which it belonged. Subsequently, these synthetic animals were added to the training set of each population, resulting in an augmented dataset. DL models were trained using the augmented and real data sets, and their prediction accuracies were compared. The VAE workflow was implemented using PyTorch (Paszke et al. 2019) enabling efficient training and the generation of synthetic data. Figure 3 provides a schematic illustration of the VAE workflow, while Supplementary Table 1 presents a summary of the relevant hyperparameters used to train the VAE model.

### Estimating the nonadditive variance captured by DL

We introduce a method to linearly approximate the model-learned genetic effects and estimate the nonadditive genetic variance captured by the DL models. To achieve this, we fed a dummy (m + 1, m) matrix (**K**) to each trained DL model. The matrix **K** consists of a vertical concatenation of a vector of zeroes and an identity matrix of SNP genotypes. The rows of **K** represent pseudo-genotype records for which model predictions are needed. The first pseudo-genotype contains a row of zeros, ie all SNPs have genotype 0, resulting in a predicted phenotype of $\hat{y_{0}}$. The second record has genotype code 1 for SNP 1 and code 0 for all other SNPs, resulting in a model prediction of $\hat{y_{1}}$. The additive effect of SNP 1 is thus $a_{1}=\hat{y_{1}}-\hat{y_{0}}$, where all other SNPs have the same genotype code (0). The additive effect of SNP 2 is obtained as $a_{2}=\hat{y_{2}}-\hat{y_{0}}$, ie the prediction from row 3 minus row one of **K**, and so on. The (additive) EBV is then obtained by multiplying the genotypes of an animal by these additive effects (a):


(13) EBV=Xa

Whereas the prediction of the (nonadditive) genetic value of the DL model is:


(14) EGV=f(X),

where *f* () denotes a trained DL model. Furthermore, we denote:


(15) $$n^{2}=1-corr(\mathrm{EGV},\mathrm{EBV}{)}^{2},$$

where $n^{2}$ is the proportion of nonadditive genetic variance relative to the total genetic variance, EGV is the estimated genetic value of each test animal for all splits, and EBV is the estimated breeding value of each test animal for all splits. The nonadditive variance estimated using DL is expressed as:


(16) $$\sigma_{NA_{DL}}^{2}=\;n^{2}*\;\sigma_{u_{DL}}^{2},$$

where $\sigma_{NA_{DL}}^{2}$ is the estimated nonadditive variance using DL, and $\sigma_{u_{DL}}^{2}$ is the genetic variance of the test population ($y_{test}$) predictions using DL.

## Results

### Additive and nonadditive genetic variance using GBLUP

Table 4 presents the proportions of additive and nonadditive genetic variances (dominance and second-order additive by additive epistasis) for FE, estimated for both populations. The narrow-sense heritability (*h*²) was estimated at 0.242 for the sire line population in both additive (A) and additive-dominance (AD) models. The sire line dominance proportion in the AD and ADE models accounted for 0.006 and 0.004, respectively. The epistasis proportion was 0.00 for both AE and ADE models. The dam line population had a similar *h*² estimate of 0.260 for both the A and AD models, as well as 0.255 for the AE and ADE models. The dominance proportion was more substantial in this population, estimated at 0.022 for the AD model and 0.015 for the ADE model and was statistically significantly greater than 0. In contrast, the epistasis proportion was small, and it was the same for both the AE and ADE models. The largest broad-sense heritability (H²) was obtained when the additive model was expanded to include dominance effects (AD model), but differences between broad and narrow-sense heritability were not statistically significant.

**Table 4.** Estimates of variance components and their ratios to phenotypic variance.

		Components							
Line	Model	$\sigma_{a}^{2}$	$\sigma_{d}^{2}$	$\sigma_{e_{aa}}^{2}$	$\sigma_{\varepsilon }^{2}$	*h* ^2^	*d* ^2^	$e_{aa}^{2}$	*H* ^2^
**Sire**	A	0.251 (0.014)	-	-	0.788 (0.010)	0.242 (0.011)	-	-	-
	AD	0.252 (0.014)	0.006 (0.004)	-	0.782 (0.011)	0.242 (0.011)	0.006 (0.004)	-	0.248 (0.012)
	AE	0.249 (0.014)	-	0.000 (0.015)	0.791 (0.016)	0.239 (0.011)	-	0.000 (0.014)	0.239 (0.017)
	ADE	0.250 (0.014)	0.004 (0.004)	0.000 (0.015)	0.785 (0.016)	0.241 (0.011)	0.004 (0.004)	0.000 (0.014)	0.245 (0.017)
**Dam**	A	0.267 (0.016)	-	-	0.761 (0.010)	0.260 (0.012)	-	-	-
	AD	0.266 (0.016)	0.023 (0.005)	-	0.737 (0.011)	0.260 (0.012)	0.022 (0.004)		0.282 (0.013)
	AE	0.262 (0.016)	-	0.000 (0.021)	0.766 (0.022)	0.255 (0.012)	-	0.000 (0.021)	0.255 (0.022)
	ADE	0.262 (0.016)	0.016 (0.004)	0.000 (0.021)	0.750 (0.022)	0.255 (0.012)	0.015 (0.004)	0.000 (0.021)	0.270 (0.022)

The results of variance components (and their standard errors) were estimated using all markers and individuals for each line. The phenotypes were standardized to have a mean of 0 and a standard deviation of 1. *h*^2^ = narrow-sense heritability, *d*^2^ = dominance ratio, $e_{aa}^{2}$ = epistasis ratio, *H*^2^ = broad-sense heritability. Standard errors are in parenthesis. A represents the animal model, gradually extended to include dominance AD, epistasis AE, and both dominance and epistasis ADE models.

### Predictive ability of linear vs DL models

Table 5 shows the predictive ability of the linear and DL models for FE in the datasets. In the sire line population, the linear genomic prediction models demonstrated consistent predictive abilities across all scenarios (A, AD, AE, ADE), achieving a correlation coefficient of 0.366. In contrast, DL models exhibited varying performances. The averaged-predictions approach of DL models achieved 0.381 and 0.377 for the MLP and CNN architectures, respectively, which are higher than those of the linear genomic prediction models and the splits-average approach of DL models. The splits-average approach of DL models yielded values of 0.367 for MLP and 0.363 for CNN, showing slightly reduced performance compared to the averaged-predictions approach but maintaining comparable predictive ability to the linear genomic prediction models.

**Table 5.** Predictive abilities of linear and DL models for predicting feed efficiency trait in the real and augmented sire and dam lines populations.

Method	Dataset	Approach	Model	Sire line	Dam line
Linear	Real	Pearson correlation	A	0.366	0.354
			AD	0.366	0.353
			AE	0.366	0.354
			ADE	0.366	0.353
DL	Real	Averaged-predictions	MLP	0.381	0.364
			CNN	0.377	0.353
		Splits-average	MLP	0.367	0.349
			CNN	0.363	0.343
	Augmented	Averaged-predictions	MLP	0.378	0.363
			CNN	0.372	0.358
		Splits-average	MLP	0.366	0.350
			CNN	0.360	0.341

The additive (A) model is equivalent to GBLUP, and it has been extended to include dominance (AD), epistasis (AE), and both dominance and epistasis (ADE). The splits-average values of DL models are the average of 50 independent partitions, while the averaged-predictions value is the per-animal average. The standard errors vary from 0.020 to 0.021 for both populations.

**Fig. 4.**
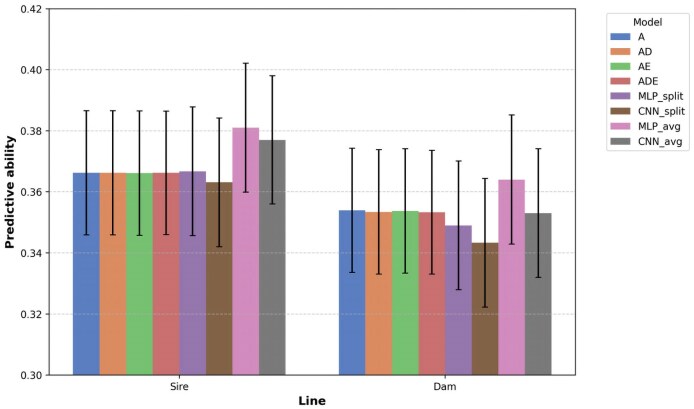
A bar plot showing the predictive ability of linear and DL genomic prediction models for the real dataset.

A similar trend was observed for the dam line dataset. The linear genomic prediction models achieved predictive abilities of 0.354 and 0.353 across the different scenarios. The averaged-predictions approach for MLP improved upon these results, while the CNN did not, with values of 0.364 for MLP and 0.353 for CNN. However, the DL splits-average approach demonstrated marginally lower performance, with predictive abilities of 0.349 for MLP and 0.343 for CNN, as shown in Fig. 4.

### Data augmentation and DL predictive ability

**Fig. 5.**
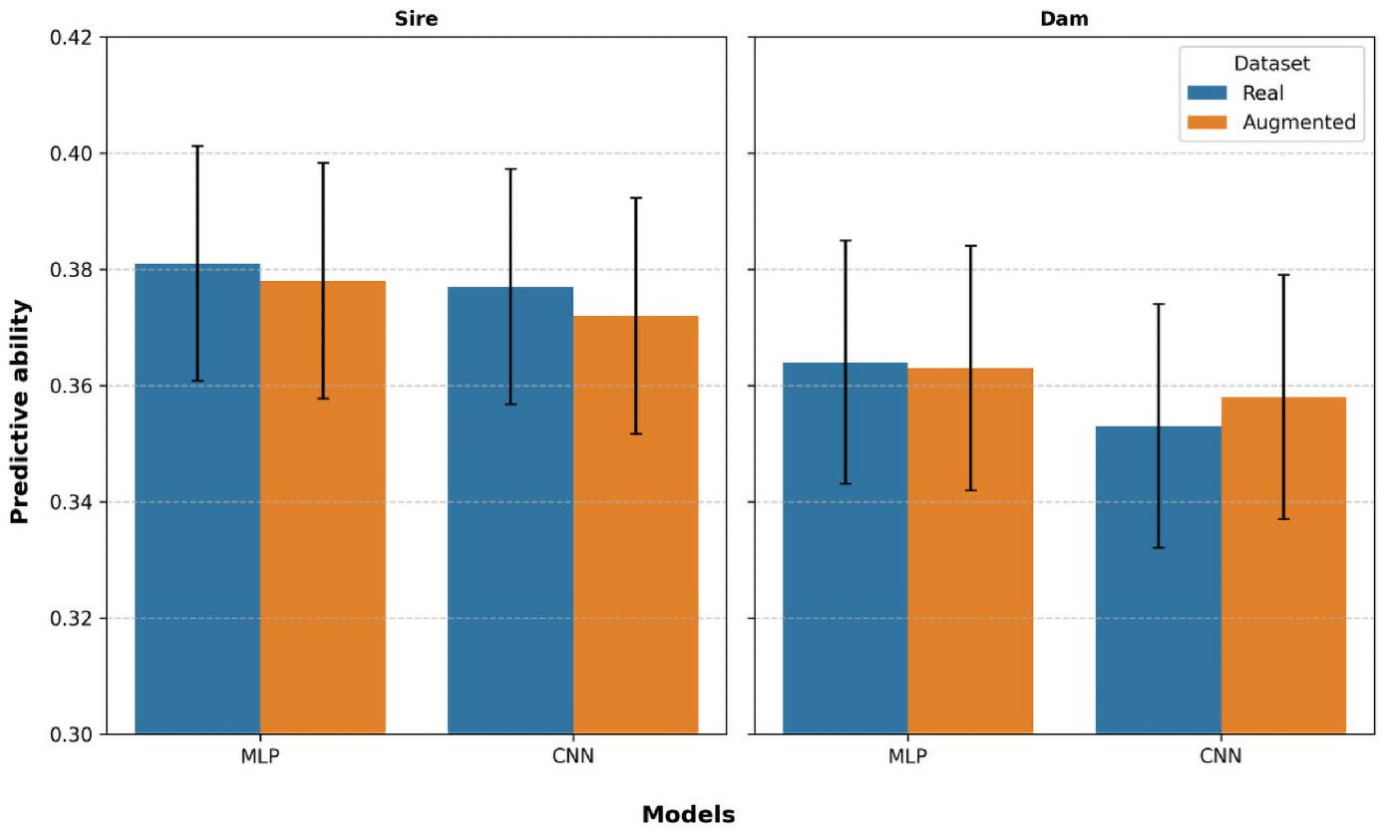
A bar plot showing predictive ability based on the averaged-predictions approach of DL models for real and augmented datasets.

The effect of data augmentation on the predictive ability of DL models was studied. In the sire line population, the averaged-predictions approach of DL models achieved values of 0.378 (MLP) and 0.372 (CNN), whereas their splits-average counterparts achieved values of 0.366 for MLP and 0.360 for CNN. Augmentation did not improve the predictive ability of DL models in this population. In the dam line population, the averaged-predictions approach attained predictive abilities of 0.363 (MLP) and 0.358 (CNN), while the splits-average approach achieved predictive abilities of 0.350 (MLP) and 0.341 (CNN). Here, augmentation slightly improved the predictive ability of the averaged-predictions approach for the CNN (from 0.353 to 0.358) and the splits-average approach for the MLP (from 0.349 to 0.350). Figure 5 presents a bar plot of the predictive ability (averaged-predictions) of the DL models for real and augmented data.

### Nonadditive genetic variance and predictive ability in DL models

Table 6 presents the proportion of nonadditive genetic variance relative to total genetic variance (*n^2^*) and the corresponding nonadditive variance ($\sigma_{NA_{DL}}^{2}$) estimated using DL models, as well as the impact of their inclusion on the predictive ability of the models. In the sire line dataset, the MLP model had a slightly higher estimated nonadditive genetic variance ($\sigma_{NA_{DL}}^{2}$ = 0.003) than the CNN model ($\sigma_{NA_{DL}}^{2}$ = 0.002). The predictive ability of both models either remained constant or improved slightly when all genetic effects were included. For the dam line dataset, the MLP model ($\sigma_{NA_{DL}}^{2}$ = 0.003) had a higher estimated nonadditive genetic variance than the CNN model ($\sigma_{NA_{DL}}^{2}$ = 0.002). The predictive ability of MLP was slightly higher when nonadditive effects were included but that of the CNN model remained unchanged.

**Table 6.** Estimated nonadditive variance by DL models and its effect on predictive ability.

Line	Model	*n^2^*	$\sigma_{NA_{DL}}^{2}$	Cor(avg_ebv, ytest)	Cor(avg_egv, ytest)
Sire	MLP	0.018	0.003	0.378 (0.020)	0.381 (0.020)
	CNN	0.014	0.002	0.377 (0.020)	0.377 (0.020)
Dam	MLP	0.017	0.003	0.363 (0.021)	0.364 (0.021)
	CNN	0.011	0.002	0.353 (0.021)	0.353 (0.021)

*n* ^2^ is the proportion of nonadditive genetic variance relative to the total genetic variance for DL models, $\sigma_{NA_{DL}}^{2}$ is the nonadditive genetic variance estimated using DL, Cor(avg_ebv, ytest) is the prediction's average correlation when only additive genetic effects of markers were multiplied by animal genotypes, while cor(avg_egv, ytest) is the prediction's average correlation when nonadditive DL models were used. The standard errors are in brackets.

## Discussion

The primary aims of this study were to evaluate and compare the predictive ability of linear and DL models, to investigate the ability of DL to capture nonadditive genetic effects for the FE trait, and to examine their impact on predictive ability. These nonadditive genetic effects have often been overlooked in breeding programs, as most breeding schemes employ genomic prediction models that utilize only additive genetic effects of markers, and breeding values are, per definition, additive effects (Falconer and MacKay 1996). However, ignoring these nonadditive genetic effects might lower the long-term genetic improvement of animal populations due to an increased loss of genetic diversity (Doekes et al. 2018). Additionally, in situations where we aim to predict total genetic effects or phenotypes, including nonadditive effects may enhance predictions. Since the underlying genetic architecture of FE is complex and unknown, we used nonparametric DL models to better model this trait. We found that the averaged-predictions approach from the MLP models improved predictive ability by approximately 4.1% (sire line) and 2.8% (dam line) for the FE trait of young animals compared to the linear genomic prediction models.

### Additive and nonadditive variance estimation using linear models

The additive and nonadditive genetic variance estimated using conventional linear models provides insight into the proportion of nonadditive genetic variance that DL models are expected to capture, as well as determining whether including these nonadditive genetic variances improves predictive ability. The proportion of additive genetic variance (heritability) for FE reported in our study was moderate and falls within the 0.13 to 0.36 range reported by Li et al. (2022) for 4 FE traits (Feed conversion ratio, residual feed intake, average daily gain, and average daily intake) in Yorkshire and Duroc boars using growth information from 30 to 100 kg body weight data. Also, Do et al. (2013), Homma et al. (2021), and Tusell et al. (2019) reported moderate heritability for feed conversion ratio and other FE traits in their study of Danish, Japanese, and French pig breeds, respectively. The differences in heritability reported by these studies may be due to several reasons, including the definition of the trait, the age of the animals, the breed, and the statistical model used.

The percentage contribution of dominance variance to total phenotypic variance, estimated for the FE trait using linear genomic prediction models in this study, was not sizable, ranging between 0.38% and 2.24%, which is lower than the 9% dominance contribution reported by Tusell et al. (2019) for feed conversion ratio. Epistasis variance (additive-by-additive) contributed nothing to the total phenotypic variance in our study, consistent with the report by Crow (2010). In fact, the inclusion of epistatic effects led to a slight reduction of the estimated additive genetic variance (de Oliveira et al. 2023) and broad-sense heritability in both populations.

The differences observed in the proportion of the additive and nonadditive variances for FE in the populations we studied may be attributed to differences in the selection focus of the lines. The sire line is strongly selected for growth and production traits, which are primarily affected by additive effects and thus have moderate to high heritability. Strong selection for these traits drives favorable alleles toward fixation, leading to a depletion of dominance variance, as they are dependent on allele frequency (Falconer and MacKay 1996). Conversely, for the dam line, focus is more on reproductive traits, including litter size, piglet survival, and mothering ability, which are mostly lowly heritable, more complex, polygenic, and more sensitive to gene interactions.

### Predictive ability of linear vs DL models

All the linear models evaluated performed similarly, and the inclusion of nonadditive genetic effects in linear genomic prediction models (AD, AE, ADE) did not improve predictive ability for the FE trait, in line with the reports of Bolormaa et al. (2015), de Oliveira et al. (2023), Varona et al. (2018), and Vitezica et al. (2018)

The predictive ability of the DL models was calculated using the averaged-predictions over split data sets and the splits-average methods. The core idea of the averaged-predictions approach is similar to the bootstrap aggregating ensemble method (Breiman 1996). This method requires that multiple models be trained on different random subsets of the training animals (with replacement) and predictions be made for the masked or held-out test animals. The average of all predictions for each test animal from all models is then compared to their true phenotype, resulting in one overall correlation metric without a natural standard deviation. Unlike the splits-average method, which provide a single standard deviation value to inform the model's robustness and variability in overall performance, the deviations obtainable for the averaged-predictions method are per-animal uncertainties and thus do not show variance across splits. An advantage of this method is that it combines predictions from multiple models to stabilize the output, thereby reducing prediction variance and yielding more stable and accurate predictions for individual samples.

As shown in Table 5, the results of the averaged-predictions method outperformed the splits-average method of DL and linear genomic prediction models in almost all scenarios. Moreover, compared to the linear genomic prediction models, the MLP model using this approach resulted in a 4.1% (sire line) and 2.8% (dam line) improvement in the predictive ability of the FE trait. The splits-average approach is the conventional method for assessing DL model performance, and it has been used by many researchers (Gianola et al. 2011; González-Camacho et al. 2012; Pérez-Rodríguez et al. 2012; Ehret et al. 2015; Abdollahi-Arpanahi et al. 2020; Pedrosa et al. 2024). Its performance is similar to, slightly higher than, or slightly lower than that of the linear models, with no significant difference as observed in our study.

Our findings are in agreement with the study by Pedrosa et al. (2024), which compared the performance of 4 genomic prediction models (Bayesian lasso, MLP, CNN, and GBLUP) for predicting behavioral traits in cows and reported superior performance of DL models. Additionally, Waldmann et al. (2020) reported in their study on the number of piglets born alive that DL models performed better than GBLUP. Contrary to our findings, Abdollahi-Arpanahi et al. (2020) compared the predictive abilities of 2 DL models (MLP and CNN), 2 ensemble learning models (random forest and gradient-boosting), and 2 parametric models (GBLUP and Bayes B) for predicting sire conception rates in bulls. They reported that the gradient-boosting model demonstrated the best predictive ability, whereas the DL models exhibited lower predictive abilities. In addition, Ehret et al. (2015) reportedly found nonrelevant differences when comparing the performance of different MLP architectures to GBLUP for predicting milk traits in Holstein and Fleckvieh bulls.

Conversely, Bellot et al. (2018) and Rachmatia et al. (2017) reported the superiority of the penalized linear model over DL models in their study of human and wheat datasets, respectively, while Montesinos-López et al. (2018) reported that GBLUP performed better than DL models when G × E interaction was taken into account in the maize and wheat datasets. The difference in the performance of the DL models reported in these studies might be due to the architecture of the trait, sample size, and hyperparameter tuning (Lourenco et al. 2024). DL models are not plug-and-play, requiring appropriate tuning to get the best performance; thus, the best hyperparameter for one population might not be the best for another (Chen et al. 2023). The activation function is one such hyperparameter. Researchers often use the ReLU activation function, which is inherently biased, particularly for regression problems where the output can range from negative to positive numbers. In such a case, the ReLu activation function will not give the best performance because, mathematically, its output can only be positive, ie max(0,x).

Furthermore, the number of hidden layers and neurons, often referred to as model depth and width, are essential hyperparameters determining how well a DL model performs (Ehret et al. 2015). Highly complex models, with multiple hidden layers and thousands of neurons, are time-consuming and tend to collapse or overfit, resulting in poor predictions. Conversely, very simple models tend not to learn the landscape of the data input and thus underfit. The choice of model also depends on the study's goal. If the goal is to predict breeding values, there might be limited benefits to using models that capture nonlinear effects, as breeding values are linear with respect to genotypes. On the contrary, when the goal is to predict the total genetic value or phenotypes of complex traits such as feed efficiency, DL models can capture nonlinear relationships adaptively and, thus, might perform better than linear models (Gianola et al. 2011).

### Data augmentation and DL predictive ability

Data augmentation using VAEs may provide a solution to the issue of limited sample size, which affects the predictive ability and estimation of genetic parameters in genomic studies, as it can be used to increase the number of observations in small populations moderately (Geleta et al. 2023). However, it is essential to emphasize that the purpose of augmentation is not to replace the real dataset; therefore, researchers should be cautious not to generate too many synthetic observations. Data augmentation with VAEs has been used to improve the performance of DL models in the domains of computer vision and natural language processing. In the genomics domain, VAEs have been used to visualize population structure from genotypes (Battey et al. 2021) and to generate artificial yet realistic genotype sequences to infer ancestry (Montserrat et al. 2019). Here, we were interested in whether augmenting our dataset with VAEs would improve the predictive performance of DL models. We increased the training population of each line by generating 1,000 synthetic observations, which were added to the real dataset and then used to train DL models. However, this did not improve the performance of our DL models.

The non-improvement of the DL models performance using the augmented dataset might be due to the availability of a sizeable amount of training data; therefore, DL models may not have benefited from augmentation. Additionally, unlike other augmentation methods, such as flipping, rotation, and cropping (which are not suited for tabular data), VAE does not introduce new variation into the dataset, as it generates new data by reconstructing the learned features from the latent space. Other factors that may affect DL model performance when using augmented data include VAE model complexity, hyperparameter tuning, and the number of synthetic data points generated. We did not explore this in-depth due to computational demands and time constraints. Future research is needed in this domain to properly adapt this method to genomic datasets.

### Nonadditive genetic variance estimated using DL models

Several researchers (Azodi et al. 2019; Pérez-Enciso and Zingaretti 2019; Abdollahi-Arpanahi et al. 2020; Costa-Neto et al. 2021) have reported that DL may capture unknown interactions between markers. Here, we introduce an approach to estimate the nonadditive genetic variance captured by DL. We also assess the contributions of this nonadditive genetic variance to the predictive ability of the FE trait in the studied populations.

It is essential to note that the estimated nonadditive genetic variance from DL models was calculated relative to the genetic variance of the model. This is because DL models do not explicitly model residual variance, making the total phenotypic variance ambiguous unless it is approximated. Therefore, a direct comparison to the nonadditive variance values from linear models, which were calculated relative to phenotypic variance, would be misleading. To ensure meaningful comparisons on a consistent scale, we rescaled the nonadditive variance estimates using linear models relative to their total genetic variances. Across linear models, the nonadditive variance accounted for about 8% (dam line, AD model) of the total genetic variance, while the estimated nonadditive variance for the DL models ranged between 1.14% and 1.85%, suggesting that DL is capable of modelling nonadditive patterns. However, the linear approximation method we introduced may not provide a complete estimate of the magnitude of the nonadditive effects it captures. These nonadditive effects had a minimal contribution to the predictive ability, in line with the reports of Crow (2010) and Vitezica et al. (2018).

Although the proportion of nonadditive genetic variance estimates in these populations was minimal, our results show that DL models were still able to capture it. Our findings are in line with recent studies of van Hilten et al. (2025) and Perelygin et al. (2025) where they showed that DL models can capture nonadditive genetic interactions, particularly epistasis. An advantage of using DL models is that it does not require the construction of several nonadditive relationship matrices, particularly when estimating epistatic effects. However, we were unable to further decouple the nonadditive genetic variance captured by DL into its dominance and epistasis components. Overall, while DL offers potential for modelling complex genetic architectures, its sensitivity to nonadditive variance may depend on network design, training strategies, and population structure.

## Conclusion

This study concludes that (i) the proportion of nonadditive variances estimated for the FE trait in the studied boar population was low. (ii) DL models, particularly MLP, had the best predictive ability for FE in our boar datasets. (iii) Data augmentation with VAE did not increase the predictive ability of DL models. (iv) DL models captured nonadditive genetic variance; however, this had minimal impact on the predictive ability. Incorporating these nonadditive genetic effects provided a slight improvement in most cases of DL models. Therefore, we recommend using DL models when total (additive and nonadditive) genetic effects are required and when phenotype prediction is the primary goal. Although this comes with significantly higher computation costs and requires rigorous model tuning.

## Supplementary Material

jkaf274_Supplementary_Data

## Data Availability

The data supporting this article are openly available in Figshare and can be found at https://dx.doi.org/10.6084/m9.figshare.30539882
