# Supplementary material for: Genomic prediction of feed efficiency in boars by deep learning
Source: G3 (Bethesda). 2025 Nov 14;16(1):jkaf274. doi: 10.1093/g3journal/jkaf274 (PMC12774587; doi:10.1093/g3journal/jkaf274)
Supplement: jkaf274_Supplementary_Data [file jkaf274_supplementary_data.docx]

**Additional file 1 Table S1:** Summary of hyperparameters used to train VAE model to generate synthentic data

| **Hyperparameter** | **Value** | **Description** |
| --- | --- | --- |
| Number of classes/bins | 10 | Number of unique classes |
| Latent dimension | 1/3 * input dimension | Size of the latent space representation. |
| Encoder layers | 4 | [96 → 64 → 32 → 16] with ReLu activations. |
| Decoder layers | 4 | [16 → 32 → 64 → 96 → output_dim] with ReLu and final Sigmoid activation. |
| Batch size | 64 | Number of samples per training batch |
| Optimizer | Adam | Optimization algorithm used for training. |
| Learning rate | 0.001 | Initial learning rate for the optimizer. |
| Loss function | Binary Cross-Entropy + KL Divergence | Combines reconstruction and regularization loss. |
| Number of epochs | 25 | Total number of passes through the training dataset. |
| Activation functions | ReLu (hidden), Sigmoid (output) | Non-linear activations used in the model layers. |

The same hyperparameters and architecture was used for Duroc and Landrace dataset
